# Quantum Monte Carlo Simulations of the Vibrational Wavefunction of the Aromatic Cyclo[10]carbon Using a Full Dimensional Permutationally Invariant Potential Energy Surface

**DOI:** 10.1021/acs.jpclett.4c00893

**Published:** 2024-05-03

**Authors:** Benjamin D. Gibbas, Martina Kaledin, Alexey L. Kaledin

**Affiliations:** † Department of Chemistry & Biochemistry, Kennesaw State University, 370 Paulding Ave NW, Box # 1203, Kennesaw, Georgia 30144, United States; ‡ Cherry L. Emerson Center for Scientific Computation and Department of Chemistry, Emory University, 1515 Dickey Drive, Atlanta, Georgia 30322, United States

## Abstract

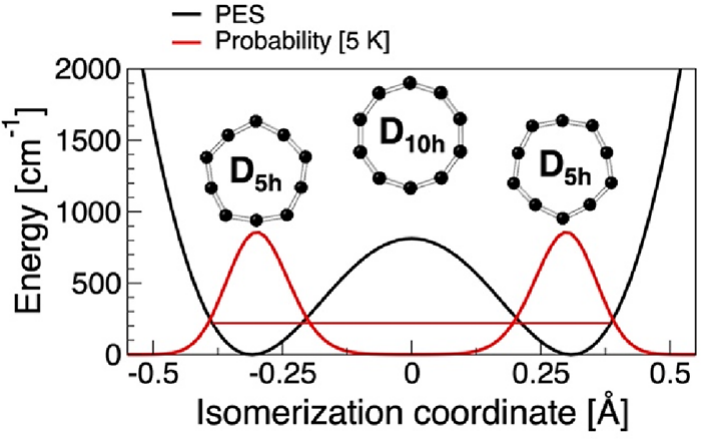

New experimental measurements [Sun et al., *Nature***2023**, 623, 972] of the cyclic C_10_ reveal a cumulenic pentagon-like D_5h_ structure at ∼5 K. However, the long-standing presumption that a large zero-point vibrational energy combined with an extremely flat D_5h_ ↔ D_10h_ ↔ D_5h_ isomerization pathway washes out the pentagonal D_5h_ structure and yields a symmetric D_10h_ decagon remains at odds with the experiment. We resolve this issue with our fitting approach based on a bond-order charge-density matrix expressed in permutationally invariant polynomials. We train the model on τHCTH/cc-pVQZ data morphed to reproduce a relativistic all-electron CCSDT(Q)/CBS D_5h_–D_10h_ potential energy barrier (benchmarked previously by others). Large scale diffusion Monte Carlo simulations in full dimensionality show that the vibrational ground state of C_10_ has compositional character of more than 96% D_5h_, fully reflecting the experimental imaging data. Quantum mechanical variational calculations in 1-D further suggest persistence of the D_5h_ symmetry structure at higher temperatures.

The family of carbon nanoclusters consists of smaller clusters, larger fullerenes, and amorphous nanometer-sized particles. These clusters have a complex structural flexibility that increases with the cluster size. Describing this structural variability and predicting stable allotropes are crucial to advancing technological applications of these materials, but it is a challenging task. Prior to the mid-1980s, it was commonly agreed that elemental carbon existed only as either a graphite or diamond structure. Knowledge of carbon allotropes was expanded upon in 1985^1^ with the discovery of an icosahedral C_60_ allotrope, commonly referred to as buckminsterfullerene. Small carbon clusters are essential in astrochemistry.^2,3^ Also, they represent intermediates in the synthesis of fullerenes^4^ and play vital roles in other material processes, including the growth of diamond thin films.^5^ Furthermore, fullerene carbon clusters have applications in multiple fields of chemistry as vessels that can hold small molecules in cages or larger molecules on its surface. This holding mechanism bears applications in medicinal and organometallic chemistry.^6^

A special class of carbon materials, the sp-hybridized cyclo[n]carbons (C_n_) consisting of two-coordinated atoms, have also been a subject of intensive research although in a single molecule environment due to their high reactivity and facile isomerization.^7−10^ Experimentally, cyclo[n]carbons with an even number of atoms are known to exist in cyclic forms in singlet electronic states and in linear forms in triplet electronic states.^11,12^ Computationally, first-principles methods, such as various pair theories, have been used to investigate clusters of smaller size,^13−19^ while the much more computationally efficient density functional theory (DFT) approaches have been used to study larger clusters.^9,20,21^ Of these, C_10_ is particularly interesting from a computational standpoint because it falls in either category, satisfies Hückel’s rules for aromaticity,^22^ adding extra stability to the cyclic conformation, and is simultaneously recognized as having an extremely complicated electronic structure.^11^

Notable first-principles and DFT studies reported over the past 30 years display an unsettling controversy regarding the identity of the cyclic cumulenic C_10_ as either D_5h_ (a quasi-pentagon) or D_10h_ (a regular decagon) structures at 0 K. Early attempts to investigate the vibrational structure of C_10_ using the Car–Parrinello molecular dynamics (CPMD) approach^23,24^ implied a rather stable D_5h_ structure up to 70 K,^24^ which was then found to become floppy by raising the temperature to 200 K and freely oscillate between the two identical pentagon-like D_5h_ minima.^23^ It is interesting to note that Andreoni et al.^23^ reported a barrier height of 806 cm^–1^ for the D_5h_–D_10h_ isomerization, which is much higher than the energy of *kT* (where *k* is the Boltzmann constant) at 200 K. This suggests that the D_5h_–D_10h_ isomerization process may not be fully understood by relying solely on the classical treatment of the vibrational dynamics of C_10_. Later computational works focused on determining the D_5h_–D_10h_ barrier height using state-of-the-art electron correlation methods. For instance, Watts and Bartlett^15^ used the frozen core (FC) CCSD(T)/[4s3p1d] level of theory to pin down the D_5h_–D_10h_ barrier at 280 cm^–1^, which is much smaller than the earlier CPMD result.^23^ Watts and Bartlett observed that due to the large zero-point vibrational energy (ZPVE) “any experimental observation of monocyclic C_l0_ will be of some averaged, effective D_10h_ structure, regardless of the detailed nature of the potential energy surface.”^15^ Van Orden and Saykally later remarked in their seminal review that “the effective vibrationally averaged structure that would be observed experimentally would be the D_10h_ ring.”^11^ Martin and Taylor,^17^ and still later Karton et al.,^18^ improved the level of electronic treatment to a “post-CCSD(T)” level in the complete basis set (CBS) limit. They claimed the D_5h_–D_10h_ barrier to be as low as 105 cm^–1^, formally confirming the previous findings based on CCSD(T) that the surface is very flat with an average structure of D_10h_ symmetry. The authors also found that the addition of the ZPVE at the harmonic level, albeit with B3LYP/cc-pVTZ, resulted in a negative barrier to pseudoinversion and, therefore, asserted that the effective C_10_ structure was a regular decagon at 0 K.^17,18^

The conclusions based on the results of the above CCSD(T) studies were rigorously questioned in a more recent and detailed investigation of the electronic structure of the cyclic C_10_ that included correlation of the 1s electrons, the full triple excitations with estimated perturbative quadruples, the CBS limit, and relativistic effects.^16^ The authors showed that by far the most important contribution to the D_5h_–D_10h_ energy difference is the inclusion of full triples in the CCSD procedure. Employing a CBS extrapolation scheme^25^ and correlating all electrons, the authors determined the D_5h_–D_10h_ electronic energy difference to be 812 cm^–1^, a benchmark to be considered definitive. Somewhat surprisingly, their barrier height falls in the range of estimates reported over the years at much lower levels of theory. Yet, estimating the effects of nuclear motion at 0 K, the authors^16^ applied a local harmonic vibrational treatment with perturbatively added elements of nonharmonic corrections along with a diagonal Born–Oppenheimer (BO) correction and, remarkably, obtained a very large correction of −777 cm^–1^. The correction is in favor of the D_10h_ structure, thus bringing the ZPVE-BO corrected D_10h_ barrier to only 35 cm^–1^ and making the two structures “practically isoenergetic”.^16^ As a result, under experimental conditions, one can expect the vibrationally averaged structure of C_10_ to resemble that of a fairly smeared-out regular decagon, consistent with the earlier computational studies.

Notwithstanding the above, there is enough reason to question the validity of the computational conclusions on several grounds: (*i*) a relatively high electronic energy barrier (812 cm^–1^), which we presently consider to be exact, (*ii*) “heavy” nuclei, (*iii*) fairly large amplitude ring-deformation motion that involves a collective coordinate, and (*iv*) a harmonic based local treatment of vibrational quantum effects. Specifically, addressing point (*iv*), we expect the inclusion of mode coupling in full dimensions and a proper treatment of large amplitude motion in a ten-atom system to introduce corrections far exceeding 35 cm^–1^. Most importantly, the present work is motivated by the recent successful synthesis of cyclo[10]carbon through dehalogenation and retro-Bergman ring-opening reaction and characterization using high-resolution atomic force microscopy.^26−28^ Therein, images of C_10_ obtained at 4.7 K showed a quasi-pentagonal cumulenic structure identified by the authors as having a D_5h_ symmetry.^26^

The aim of the present letter is 2-fold: (1) to verify the new experimental finding on the contentious issue of the vibrational symmetry of C_10_ using first-principles methods in both electronic structure theory and quantum treatment of vibrational dynamics on an analytical potential energy surface (PES), and (2) to test a new approach to learning the PES with the method of permutationally invariant polynomials (PIP)^29^ based on the bond-order charge-density matrix formalism introduced by us previously.^30^ The latter method was originally designed as a means of lowering the order of the PIP for the more challenging cases of high permutational symmetry, with C_10_ being an excellent case study. Alternative approaches aimed at using low-order polynomial regressions in representing molecular PESs in progressively higher dimensions are being actively examined by others.^31,32^

Briefly, in our approach the PES is modeled after the Hartree–Fock expression for the electronic energy: *E*_0_ = *tr*[**P**(**H** + (**G**{**P**})/2)], where **P** is the bond-order charge-density matrix, **H** is the one-electron core Hamiltonian matrix, and **G** is the two-electron part of the Fock matrix.^33^ A correction term is added as the correlation energy along with the nuclear repulsion energy,^30^
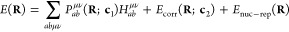
1**R** is the 3*N* Cartesian vector, **c**_1_ and **c**_2_ are the linear parameters, and *a* and *b* are atomic centers, which also serve as basis function indices in conjunction with the respective angular momentum labels μ, ν. Here, as in the preceding work,^30^ we neglect the two-electron part of the Fock matrix due to its dependence on **P**, as our present goal is to use a linear regression for PES learning. The matrix elements *H*_*ab*_^*μν*^ are the one-electron integrals known analytically as is the nuclear repulsion energy *E*_nuc–rep_. The unknown factors are the parametrized density matrix elements *P*_*ab*_^*μν*^ and the *E*_corr_ correction. Both quantities are expected to be slowly varying functions of the internuclear distances,^30^ and will be learned by fitting eq 1 to data derived from *ab initio* calculations. As an aside, since analytical gradient has not yet been implemented, the PES represented by eq 1 is currently best suited for quantum simulations, i.e., grid integrable or Monte Carlo based methods, as will be described below. In perspective, however, and as noted recently by method development groups,^34^ analytical gradients of PIP based PESs may be readily computed by the PESPIP software.

Presently, our training set generation strategy is defined by the computational complexity of the electronic structure of C_10_. It is clear that procuring thousands of all-electron calculations (all in C_1_ symmetry) at the CCSDT(Q)/CBS level to train a model with ∼1000 parameters (see below) is manifestly not feasible. Similarly, employing Δ-learning,^36^ which can normally reduce the number of expensive high-level data by a significant factor, is still computationally impractical within any reasonable time window, cf. the discussion in the preceding work.^16^ Yet, noticing that CPMD, a pseudopotential-based nongradient-corrected functional,^23^ appears to recover the D_5h_–D_10h_ barrier very close to the “exact” value,^16^ even if fortuitously, hints at certain solutions. The most important of those has been advocated by Huang et al.^37^ regarding the central role of DFT in machine learning. One possibility, thus, is to use a GGA functional (hybrid or pure) with a high-quality basis set while treating all 60 electrons explicitly. To this end, relying on the work of others^18^ and carrying out our own exploratory calculations with relevant data summarized in the SI, we observe that B3LYP^38^/cc-pVQZ overestimates the barrier by a respectable 46% and underestimates the smaller of the D_5h_ angles by 3.3° (as illustrated in Table 1). Alternatively, Handy’s kinetic-energy-corrected *meta*-GGA τHTCH functional^39^ with the cc-pVQZ basis performs substantially better, underestimating the barrier by only 8% while underestimating the corresponding angle by 2.3°. Similarly, the gradient-corrected PBE functional^40^ with the same basis underestimates the barrier by 42% but reproduces the D_5h_ structure impressively closely, cf. Table 1. Any of the above functionals can be used to train the model with appropriately applied scaling, as has been done in the past.^41−43^ Here, we have found it most practical to use τHCTH/cc-pVQZ as the source of training the PES, considering that its barrier is closest to the CCSDT(Q)/CBS benchmark value of 812 cm^–1^, and then “gently” morph the energies in the vicinity of the D_5h_ minimum to bring the D_10h_–D_5h_ difference exactly to the benchmark value. We refer to this level of theory by τHCTH-CC and use the following morphing function
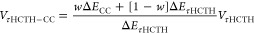
2where the zero of the *V*_τHCTH_ energy is set at the D_10h_ transition state, Δ*E*_CC_ is the benchmark (CC = CCSDT(Q)/CBS) barrier height,^16^ Δ*E*_τHCTH_ is the τHCTH barrier height, and the energy-dependent weight factor is
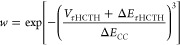
3For the geometries where *V*_τHCTH_ is slightly above the minimum, that is, where *V*_τHCTH_ ≈ −Δ*E*_τHCTH_, we have *w* ≈ 1, and therefore *V*_τHCTH-CC_ ≈ (Δ*E*_CC_/Δ*E*_τHCTH_)*V*_τHCTH_, a simple scalar modification of the original τHCTH data. At moderate and much higher energies, *w* → 0 very rapidly, given the cubed exponential, and *V*_τHCTH→CC_ ≈ *V*_τHCTH_, i.e., it remains unmodified from the τHCTH data. We also point out that the ratio Δ*E*_CC_/Δ*E*_τHCTH_ should not deviate significantly from unity to avoid introducing artificial lobs and valleys in the modified energy landscape, which can be readily seen by analyzing eq 2 in one dimension. Thus, the present choice of τHCTH/cc-pVQZ for the source data is well justified (cf. ΔΔ*E* values in Table 1).

**Table 1:** Summary of the Cumulenic D_10h_ Transition State and D_5h_ Global Minimum Structures Calculated Using Various Methods^a^

				ω	C=C	C=C	α_1_
	Δ*E*	ΔΔ*E*	Δ*H*^0^	D_10h_	D_10h_	D_5h_	D_5h_
B3LYP/cc-pVQZ	1184	146%	815	376*i*	1.2816	1.2876	126.5
τHCTH/cc-pVQZ	746	92%	413	313*i*	1.2853	1.2902	127.5
PBE/cc-pVQZ	472	58%	188	274*i*	1.2906	1.2947	129.1
(FC)-CCSD(T)/cc-pVTZ^b^	279	34%	–196	409*i*	1.2944	1.2976	128.2
(FC)-CCSD(T)/cc-pVQZ^c^	115	14%	<0	N/A	1.2914	1.2940	129.8
(FC)-CCSD(T)/CBS^d^	105	13%	<0	N/A			
CCSDT(Q)/CBS^e^	812	100%	35	N/A			

a The value of the isomerization barrier energy is Δ*E* (in cm^–1^) and its fractional value of the benchmark CCSDT(Q)/CBS energy of 812 cm^–1^ is ΔΔ*E* (in %). The ZPVE corrected barrier is Δ*H*^0^ (in cm^–1^), and the D_10h_ transition state frequency is ω (cm^–1^). The structural parameters are the C=C bond length (in Å) and the smaller of the two angles, α_1_ (in deg). At the D_10h_ transition state, angles α_1_ = α_2_ = 144°, and in the D_5h_ minimum structure, α_1_ + α_2_ = 288°. (Further details are given in the SI.)

b This work: frozen core (FC) geometry optimization and a normal-mode analysis using CFOUR.^35^

c Ref. (18): A fully optimized (FC)-CCSD(T)/cc-pVQZ geometry.

d Ref. (18): A single point (FC) energy calculation at the (FC)-CCSD(T)/cc-pVQZ optimized geometry.

e Ref. (16): A relativistic all-electron calculation at the (FC)-CCSD(T)/cc-pVQZ optimized geometry.

The result of applying eq 2 is shown in Figure 1 compared with the original τHCTH data. By design, the two curves converge to 0 at the D_10h_ transition state structure, defined by the inversion coordinate X1 = 0. The curves begin to deviate as X1 increases, and then they reach the largest gap of 66 cm^–1^ at the D_5h_ minimum (X1 = 0.31 Å), where the τHCTH-CC curve reproduces the CCSDT(Q)/CBS benchmark value of 812 cm^–1^. As X1 increases, the two curves merge as prescribed by the morphing function. Only the energies below the D_10h_ transition state are modified, while the energies above this threshold virtually coincide with the original τHCTH data. Full details on generating the training set are described in the SI.

**Figure 1:**
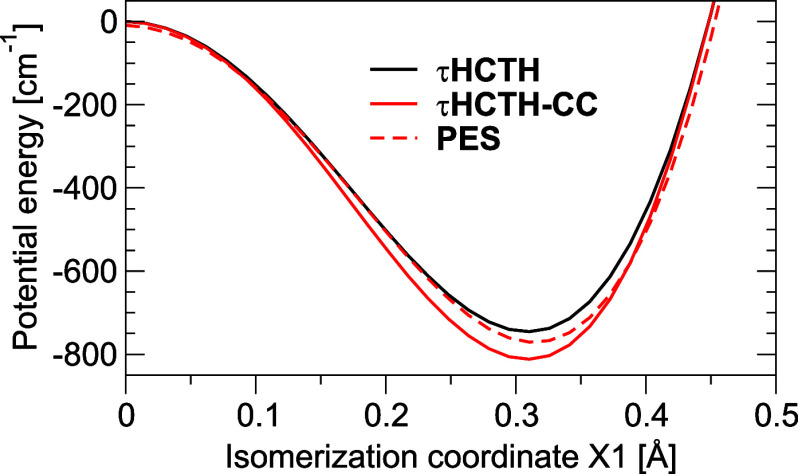
Potential energy profile along the D_5h_–D_10h_(X1 = 0) inversion coordinate calculated with τHCTH/cc-pVQZ is presented as a black curve. The τHCTH/cc-pVQZ energy morphed with eq 2 and labeled τHCTH-CC is colored solid red. The energy of the trained PES fitted to the 5500 points of τHCTH-CC data is shown as a dashed red curve. The coordinate X1 is defined as the Cartesian vector difference between the D_10h_ (the origin: X1 = 0) and one of the two D_5h_(X1 = ±0.31 Å) configurations, being oriented in the same spatial reference frame.

To train the PES defined by eq 1 and to formally represent the core Hamiltonian, we use a Cartesian Gaussian basis
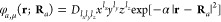
4with **r** and **R**_*a*_ being electronic and nuclear coordinates, respectively. *D*_*l*_*x*_*l*_*y*_*l*_*z*__ is the normalization constant and *l*_*x*_, *l*_*y*_, *l*_*z*_ are the orbital quantum number integers represented by μ, e.g., μ = 000 for *s*, μ = 100 for *p*_*x*_, etc. Presently, we do not distinguish between 1s and 2s electrons, and thus, for a second-row atom, an *s*-function in our definition implies a collective 1s2s AO contribution to the density matrix, resulting in a virtual C atom with four *s*-electrons and two *p*-electrons. With this definition, for a given PIP term, the number of density matrix elements *P*_*ab*_^*μν*^ is 26, i.e., 10 unique *P*_*aa*_^*μν*^ elements originating from the self-atom terms: (*ss*, *sx*, *sy*, *sz*, *xx*, *xy*, *xz*, *yy*, *yz*, *zz*), and 16 *P*_*a*≠*b*_^*μν*^ elements originating from the atom–atom terms, in a matrix representation: (*s*, *x*, *y*, *z*)(*s*, *x*, *y*, *z*)^*T*^. An additional PIP is added to train *E*_corr_, an AO independent function that mimics correlation energy, and a standalone parameter to adjust the energy origin. The PES was trained on 5500 τHCTH-CC energies with the additional constraint that the total number of electrons is 60, a requirement necessary for keeping the individual density elements less oscillatory. The latter constraint for the number of electrons *n* at a configuration **R** was enforced by the trace condition,
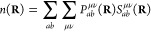
5where *S*_*ab*_^*μν*^ is the overlap. We assigned a weight of 0.001 to *n*(**R**) while assigning a weight of 1.0 to the corresponding energy thus formally constructing a weighted (110000 × (27*L* + 1)) linear system where *L* is the number of nonconstant terms in the PIP of a given degree. We generated a complete PIP of the total degree four, which for a system of 10 identical nuclei has *L* = 35, resulting in a (110000 × 946) linear system. Details of PIP composition are given in the SI. The variational coefficients **c**_1_ and **c**_2_ describing *P*_*ab*_^*μν*^ and *E*_corr_, respectively, are found by a linear regression using a QR decomposition^44^ as implemented in the DGELS subroutine supplied within the MKL software suite. Additional optimization of the Gaussian orbital exponent α and other nonlinear parameters used in the model is performed using a simplex search.

The results of training the model are presented in Figure 2 with corresponding testing results provided in the SI. There are essentially three regions of configurations, as seen in reference to the horizontal axis. They include (1) the very low energy region clustered around the D_5h_ and D_10h_ structures on the [0, 2000] cm^–1^ range; (2) the middle (bulk) region [2000, 8000] cm^–1^ describing an NVE phase space at the ZPVE energy; and (3) the sparse sample of points on the [8000, 16000] cm^–1^ range extending the PES up to the higher energies accessible in quantum simulations at 0 K. The error of the fit stays bound on roughly the [−100, 100] cm^–1^ energy span, and the total RMSE on the energy is 21 cm^–1^ or ∼0.4%. (We note in passing that the total number of electrons in the trained model, *n*(**R**), is conserved to within 0.05 electrons, or 0.08% of the total population.) The RMSE curve converges at ∼6000 cm^–1^ suggesting that the model is rather robust and apparently capable of describing both low and high energy configurations equally well. A more detailed inspection of the PES, along the D_5h_–D_10h_ inversion coordinate ,shows, as captured in Figure 1, that the trained PES passes roughly between the original and the morphed curves and somewhat underestimates the barrier height. Optimization of the geometry on the PES gives the D_10h_ C=C bond distance of 1.2845 Å and the corresponding D_5h_ distance of 1.2905 Å with the α_1_ angle of 127.9°, closely recovering the τHCTH/cc-pVQZ optimized parameters, and the calculated PES barrier height is 754 cm^–1^.

**Figure 2:**
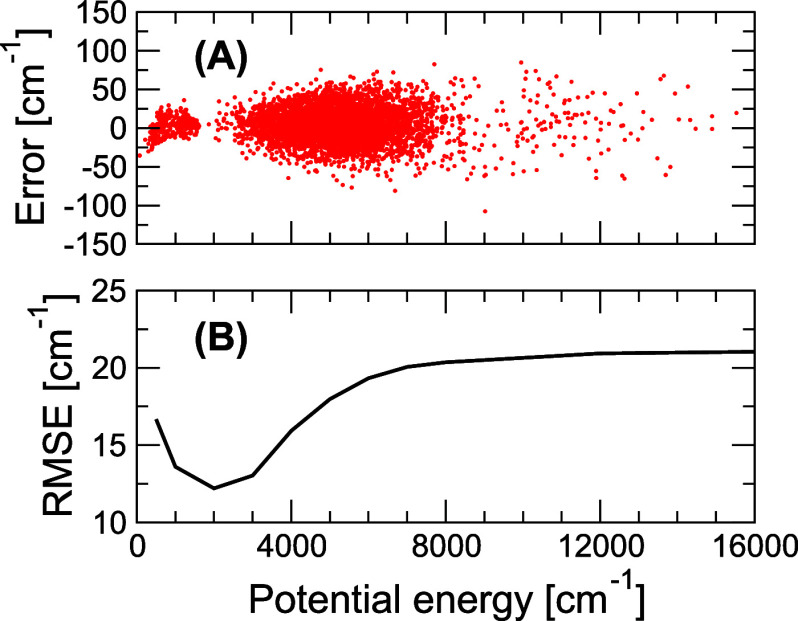
(A) The absolute error of the model trained on the τHCTH-CC data derived from 5500 configurations. (B) The cumulative RMSE of the same model as a function of the potential energy relative to the D_5h_ minimum.

Using the PES to carry out extensive diffusion Monte Carlo (DMC) simulations, additionally expounded in the SI, we took ∼30000 configurations from an equilibrated distribution to calculate a C–C distance spectrum by measuring each of the 45 internuclear distances at these configurations and binning them on the [1, 4.5] Å range. An unmistakable signature of an average D_5h_ structure is the broad but distinct double peak on [2.1, 2.8] Å (Figure 3A) corresponding to the nearest C^i^–C^i^ (innermost carbons, cf. Figure S1 for definitions) distances forming the smaller red pentagon and the nearest C^o^–C^o^ (outermost carbons) distances forming the larger green pentagon, as emphasized in Figure 3B, inset scheme. If the average structure were D_10h_, the two peaks would merge to form a single broad peak at ∼2.45 Å and the red and green pentagons would be identical. Incidentally, the spectral feature on [3, 4.5] Å is relatively insensitive to the overall structure and is not a reliable differentiator of D_5h_/D_10h_. Analyzing the spectrum in detail by fitting Gaussian functions (6 Gaussians in total, shown in Figure 3B) gives the ZPVE averaged C=C cumulenic bond of 1.298 Å, which is 0.014 Å greater than the PES equilibrium value and with a standard deviation of 0.058 Å. The C^i^–C^i^ curve (the red peak/pentagon in Figure 3B) has a 2.320 Å average (2.314 Å at the PES D_5h_ minimum) and a standard deviation of 0.084 Å. Its counterpart C^o^–C^o^ distance (the green peak/pentagon in Figure 3B) is 2.549 Å on average (2.543 Å at the PES D_5h_ minimum) with a 0.072 Å standard deviation. Using these parameters, we can derive the two ZPVE averaged angles as 126.6° and 157.9°, both smaller than their corresponding PES optimized counterparts of 127.9° and 160.1°, while adding up to 284.5°, a 3.5° deficit from the exact sum of 288°. The latter is readily explained by deviations from planarity at 0 K, which in the present DMC simulations corresponds to the ground state vibrational energy (ZPVE) of 10162 ± 53 cm^–1^.

**Figure 3:**
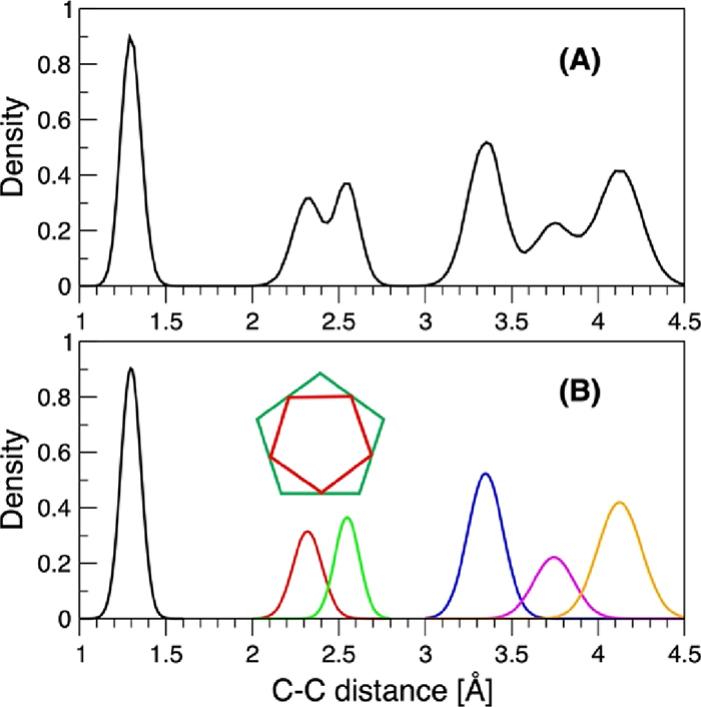
(A) C_10_ ground vibrational state C–C distance spectrum obtained from a 30000 “walker” DMC simulation. (B) A least-squares-optimized decomposition of the spectrum using floating Gaussians. The centers of the Gaussians corresponding to the six distinguishable peaks are as follows: the C=C double bond is 1.298 Å (black); the shortest innermost C^i^–C^i^ and outermost Carbon C^o^–C^o^ distances are 2.320 Å (red) and 2.549 Å (green), respectively; and the distances involving C-c-c-C, C-c-c-c-C and C-c-c-c-c-c-C centers are 3.349 Å (blue), 3.745 Å (magenta), and 4.125 Å (orange), respectively. The schematic double pentagon inset demonstrates a visual correspondence with the red and green peaks, proving the D_5h_ symmetry of the cyclic C_10_ derived from the ground state vibrational density.

The visible overlap of the C^i^–C^i^ and C^o^–C^o^ density curves, exposed in Figure 3B, raises a question on sampling of configurations where the red and green pentagons are identical, i.e., structures with the D_10h_ symmetry. The crossing point occurs at 2.439 Å, which is almost exactly the C^i^–C^i^/C^o^–C^o^ distance of 2.443 Å at the PES optimized D_10h_ structure. To make a quantitative estimate of the D_5h_/D_10h_ relative population in the ground state, we project the DMC distribution onto a pentagon–decagon inversion coordinate. For each DMC configuration, we take the difference in the innermost and outermost radii, relative to the centroid, averaged over the C_10_ ring. We call it coordinate X2 to differentiate from the linear coordinate X1 defined in Figure 1 and suitable for variational calculations. The X2-projected density is strongly localized at the D_5h_ minima with X2 = ±0.198 Å, very close to the ones reported by Andreoni et al.^23^ who used the same definition of X2. If we define a D_10h_ structure as one localized on X2 = [−δ, δ] for some small value of δ and integrate the projected density, we can estimate the relative probability of finding C_10_ in a D_5h_ or D_10h_ structure. For instance, setting δ = 0.05 Å corresponds to a 4.7° maximal deviation from the regular decagon angle, which may still be reasonably viewed as a D_10h_ structure. In this definition, the ground state composition is {99.3% D_5h_ + 0.7% D_10h_}, i.e., interpretable as being completely dominated by D_5h_. Extrapolating further to δ = 0.1 Å, which is a 9.5° maximal deviation from the characteristic D_10h_ angle, the composition becomes {95.9% D_5h_ + 4.1% D_10h_}. In other words, with a high degree of confidence, we may conclude that C_10_ at 0 K is ∼96% D_5h_ in character. A similar conclusion was made based on a 1-D variational quantum treatment of the double well potential and is summarized in the SI. The ground state was found in a deep tunneling regime of ∼200 cm^–1^ above the D_5h_ minimum or ∼611 cm^–1^ below the D_10h_ transition state with a numerically negligible probability of finding C_10_ in the D_10h_ configuration. Estimation of temperature effects suggests an extremely stable D_5h_ configuration up to 300 K beyond which the D_10h_ configuration gradually emerges as viable.

In summary, we have demonstrated via quantum vibrational Monte Carlo simulations on a full dimensional permutationally invariant PES, trained by τHCTH/cc-pVQZ data and morphed to reproduce a key CCSDT(Q)/CBS benchmark,^16^ that the cyclic C_10_ cumulene possesses a true D_5h_ quasi-pentagonal structure at 0 K, in agreement with a recent experiment,^26^ and likely thermally stable at much higher temperatures based on DVR calculations in 1-D. Our finding rests entirely on the benchmark, which predicts a high enough barrier to the D_5h_–D_10h_–D_5h_ symmetric isomerization reaction, much higher than the previously accepted range of values obtained by various authors over the past three decades using the highly respectable CCSD(T)/cc-pVTZ level of theory and its equivalents. In short, for certain classes of systems with particularly complicated electronic structure, such as that exhibited by C_10_ and ostensibly other cyclic carbon clusters, the inclusion of full triples and possibly core–valence interaction into the CCSD procedure while treating the vibrational problem quantum mechanically in full dimensions may in fact be necessary for a correct description of subtle dynamical processes.

## Supporting Information Available

Structural conventions and DFT explorations; definition of PIPs; training the model; the DMC calculations; and the DVR calculations in 1-D (PDF)
Transparent Peer Review report available (PDF)
