# Supplementary material for: Quantum Monte Carlo Simulations of the Vibrational Wavefunction of the Aromatic Cyclo[10]carbon Using a Full Dimensional Permutationally Invariant Potential Energy Surface
Source: J Phys Chem Lett. 2024 May 3;15(19):5070–5. doi: 10.1021/acs.jpclett.4c00893 (PMC11103689; doi:10.1021/acs.jpclett.4c00893)
Supplement: Supplementary file 1 — jz4c00893_si_001.pdf [file jz4c00893_si_001.pdf]

# Supporting Information: Quantum Monte Carlo Simulations of the Vibrational Wavefunction of the Aromatic Cyclo[10]carbon Using a Full Dimensional Permutationally Invariant Potential Energy Surface

*Benjamin D. Gibbas[1], Martina Kaledin,[1,*] Alexey L. Kaledin[2,*]*

[1] *Department of Chemistry & Biochemistry, Kennesaw State University, 370 Paulding Ave NW, Box # 1203, Kennesaw, Georgia 30144, United States*
[2] *Cherry L. Emerson Center for Scientific Computation and Department of Chemistry, Emory University, 1515 Dickey Drive, Atlanta, Georgia 30322, United States*

**S-1. Structural Conventions and DFT Explorations..............................................S2**

**S-2. Definition of PIPs.........................................................................................S3**

**S-3. Training the Model........................................................................................S5**

**S-4. The DMC Calculations...................................................................................S6**

**S-5. The DVR Calculations in 1-D.......................................................................S9**

**References..........................................................................................................S11**

## S-1. Structural Conventions and DFT Explorations

Figure S1 depicts the τHCTH/cc-pVQZ optimized geometry of the cyclic cumulenic $C_{10}$ in its global $D_{5h}$ minimum. Following Watts and Bartlett,[1] the superscripts "i" and "o" stand for the innermost and outermost carbons relative to the centroid, respectively.

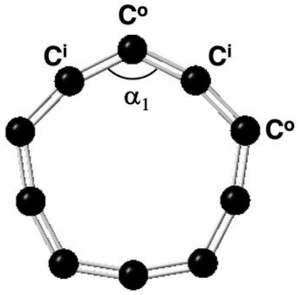


**Figure S1**. Definition of atom classes in a cumulenic (all C=C double bonds are identical) $D_{5h}$ global minimum of $C_{10}$. For this structure, $\alpha_1$ is defined as a $C^i$-$C^o$-$C^i$ angle and is related to its adjacent $\alpha_2$ = $C^o$-$C^i$-$C^o$ angle by the $\alpha_1 + \alpha_2 = 8\pi/5$ relationship.

**Table S1. Additional data on the Cumulenic $D_{10h}$ Transition State and $D_{5h}$ Global Minimum Structures Calculated Using Various DFT Levels of Theory**[a]

| | $\Delta E$ | $\Delta\Delta E$ | $\Delta H^0$ | ω $D_{10h}$ | C=C $D_{10h}$ | C=C $D_{5h}$ | $\alpha_1$ $D_{5h}$ |
|---|---|---|---|---|---|---|---|
| B3LYP/ aug-cc-pVTZ | 1268 | 156% | 866 | 396*i* | 1.2821 | 1.2883 | 126.2 |
| B971/aug-cc-pVTZ | 1787 | 220% | 1357 | 422*i* | 1.2849 | 1.2924 | 124.4 |
| B971/cc-pVQZ | 1723 | 212% | 1315 | 410*i* | 1.2844 | 1.2918 | 124.5 |
| PBE/aug-cc-pVTZ | 530 | 65% | 218 | 289*i* | 1.2910 | 1.2953 | 128.6 |
| M06L/aug-cc-pVTZ | 1371 | 169% | 915 | 424*i* | 1.2804 | 1.2873 | 125.3 |
| M06L/cc-pVQZ | 1312 | 162% | 973 | 396*i* | 1.2801 | 1.2869 | 125.6 |
| τHCTH/aug-cc-pVTZ | 787 | 97% | 439 | 323*i* | 1.2855 | 1.2903 | 127.6 |
| τHCTH/aug-cc-pVQZ | 720 | 89% | 395 | 307*i* | 1.2851 | 1.2903 | 127.6 |
| τHCTHhyb/aug-cc-pVTZ | 1475 | 182% | 1063 | 403*i* | 1.2848 | 1.2918 | 125.0 |
| τHCTHhyb/cc-pVQZ | 1419 | 175% | 1038 | 383*i* | 1.2845 | 1.2914 | 125.2 |

[a]The value of the isomerization barrier energy is $\Delta E$ (in $cm^{-1}$) and its fractional value of the benchmark CCSDT(Q)/CBS energy of 812 $cm^{-1}$ (see Table 1 of the main text) is $\Delta\Delta E$ (in %). The ZPVE corrected barrier is $\Delta H^0$ (in $cm^{-1}$), the $D_{10h}$ transition state frequency is ω ($cm^{-1}$). The structural parameters are the C=C bond lengths (in Å) and the smaller of the two $C^i$-$C^o$-$C^i$ angles $\alpha_1$ (in deg). In the $D_{10h}$ transition state, the two angles are $\alpha_1$=$\alpha_2$=144 deg, while in the $D_{5h}$ minimum structure, $\alpha_1$+$\alpha_2$=288 deg.

**S-2. Definition of PIPs**

As has been ours and other authors' practice in the past,[2-4] we recognize the importance of like nuclei permutational invariance in vibrational dynamics and point out that a PES symmetrized in such a manner not only possesses physically correct properties for like nuclei permutations but also relies on substantially fewer parameters than an unsymmetrized PES does and, incidentally, can be efficiently learned with relatively smaller sets of *ab initio* data. The latter is particularly important in the case of $C_{10}$, a computationally challenging system that requires a very high level of electron correlation.[5] Furthermore, noting the challenges of the PIP representation for systems of high permutational symmetry where monomial symmetrization effort scales with the number of like nucleus permutations (there are 10! = 3.6 * $10^6$ identical minima in $C_{10}$), and the number of polynomial terms grows extremely fast with the PIP degree.[6] We aim to represent the PES using a low-order PIP without sacrificing the accuracy of the fit. We demonstrate that this can be achieved by the approach of the bond order charge-density matrix we introduced recently for an *s*-function density.[4] Here, we expand this formulation to *sp*-function density with highly encouraging results.

Each of the PIP terms is a simple sum of so-called unsymmetrized monomials, i.e., products of internuclear distance functions $y_{ab} = exp(-r_{ab}/r_0)$, with the largest sum consisting of 37800 elements of the type $y_{ab}y_{cd}y_{ef}y_{gh}$ representing four-term quartic contributions. We use two sets of internuclear distance functions for PIPs to separate the ranges of $P_{ab}^{\mu\nu}$ and $E_{\text{corr}}$, with range parameters $r_0$ and $t_0$, respectively. Thus, training of the PES in Eq. 1 by standard linear regression is performed for each set of the three exponential parameters on a grid to minimize the RMSE.

**Table S2**. **The Composition of a Complete 4th-order PIP for 10 Identical Nuclei**.

| PIP number | monomials in PIP | functions in monomial | monomial power composition |
|---|---|---|---|
| 1 | 1 | 1 | 0 |
| 2 | 45 | 1 | 1 |
| 3 | 45 | 1 | 2 |
| 4 | 45 | 1 | 3 |
| 5 | 45 | 1 | 4 |
| 6 | 360 | 2 | 1 + 1 |
| 7 | 630 | 2 | 1 + 1 |
| 8 | 360 | 2 | 2 + 2 |
| 9 | 630 | 2 | 2 + 2 |
| 10 | 720 | 2 | 1 + 2 |
| 11 | 1260 | 2 | 1 + 2 |
| 12 | 720 | 2 | 1 + 3 |
| 13 | 1260 | 2 | 1 + 3 |
| 14 | 3150 | 3 | 1 + 1 + 1 |
| 15 | 7560 | 3 | 1 + 1 + 1 |
| 16 | 2520 | 3 | 1 + 1 + 1 |
| 17 | 120 | 3 | 1 + 1 + 1 |
| 18 | 840 | 3 | 1 + 1 + 1 |
| 19 | 9450 | 3 | 1 + 1 + 2 |
| 20 | 15120 | 3 | 1 + 1 + 2 |
| 21 | 7560 | 3 | 1 + 1 + 2 |
| 22 | 2520 | 3 | 1 + 1 + 2 |
| 23 | 5040 | 3 | 1 + 1 + 2 |
| 24 | 360 | 3 | 1 + 1 + 2 |
| 25 | 2520 | 3 | 1 + 1 + 2 |
| 26 | 4725 | 4 | 1 + 1 + 1 + 1 |
| 27 | 37800 | 4 | 1 + 1 + 1 + 1 |
| 28 | 18900 | 4 | 1 + 1 + 1 + 1 |
| 29 | 37800 | 4 | 1 + 1 + 1 + 1 |
| 30 | 15120 | 4 | 1 + 1 + 1 + 1 |
| 31 | 630 | 4 | 1 + 1 + 1 + 1 |
| 32 | 2520 | 4 | 1 + 1 + 1 + 1 |
| 33 | 12600 | 4 | 1 + 1 + 1 + 1 |
| 34 | 15120 | 4 | 1 + 1 + 1 + 1 |
| 35 | 2520 | 4 | 1 + 1 + 1 + 1 |
| 36 | 1260 | 4 | 1 + 1 + 1 + 1 |

**S-3. Training the Model**

We generated a set of nuclear configurations by "on-the-fly" propagating several sets of NVE trajectories using a low level of theory τHCTH/cc-pVTZ'(without the *f*-functions) level of theory with a velocity-Verlet integrator step of 1 fs with one energy and one gradient calculation per step. The sets were split into two approximately equal parts for training and testing the model and were generated as follows: (**SET-1**) the bulk of the training set contains 5000 points, one saved every 2 fs, along a trajectory with the total energy corresponding to the harmonic ZPVE of 10500 $cm^{-1}$; (**SET-2**) the $D_{5h}$ vicinity set contains 100 points, one saved every 2 fs, from a trajectory with a total energy of 1000 $cm^{-1}$ above the $D_{5h}$ global minimum; (**SET-3** and **SET-4**) two sets, each of 100 points and saved every 2 fs, "anchored" around the $D_{10h}$ structure by adding a symmetric harmonic potential to the τHCTH/cc-pVTZ' potential with a tight and a loose force constant to sample the region in the $D_{10h}$ structure vicinity; (**SET-5**) a sparse, high energy set of 200 points produced from the total energy of 2 * ZPVE = 21000 $cm^{-1}$ by saving every 10 fs generated for potential use in finite temperature quantum (path integral) simulations. The full training set thus contains 5500 configurations describing all relevant isomers over a reasonably wide potential energy range: 0 – 16000 $cm^{-1}$. As a next step, the selected configurations were used to calculate the energies with the higher quality τHCTH/cc-pVQZ level of theory before applying the morphing procedure described above. The training and test errors of the low-level data are illustrated in Figure S2. As can be seen, the model performs quite well and appears to avoid overfitting problems, at least in the vicinity of **SET-1** configurations.

The above electronic structure calculations were done with Gaussian[7] and MOLPRO programs[8].

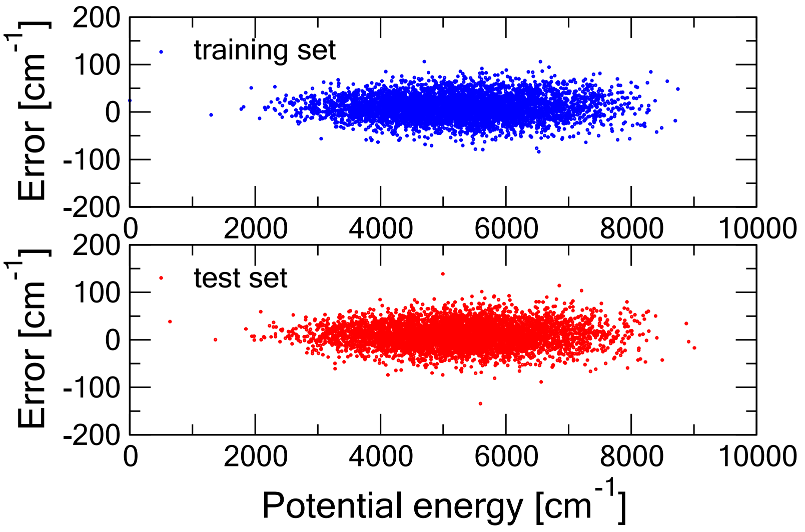


**Figure S2**. Errors of the model trained on the low-quality τHCTH/cc-pVTZ' training set **SET-1** (blue) and tested on the complement of **SET-1** (red) as functions of the potential energy above the $D_{5h}$ global minimum. The respective RMSEs are 26.5 and 26.9 $cm^{-1}$. The optimized non-linear parameters are: $r_0$ = 3.5 bohr, $t_0$ = 2.0 bohr and $\alpha$ = 0.1 $bohr^{-2}$.

**S-4. The DMC calculations**

The trained PES is used in a multi-step DMC procedure, which we implemented following the original prescription of Anderson.[9] We carried out the simulation in several stages. First, starting at the delta function distribution of 1000 walkers at the $D_{10h}$ configuration, we briefly propagated the initial packet with a time step of 3 a.u. until a target distribution of 30000 walkers was gradually reached. Subsequently, we equilibrated that distribution with a time step of 1 a.u. for 500 steps before decreasing the propagation step to 0.5 a.u. to generate a well-equilibrated distribution following an 800 steps run, shown in Figure S3, followed by additional statistical data in Figure S4 and Figure S5.

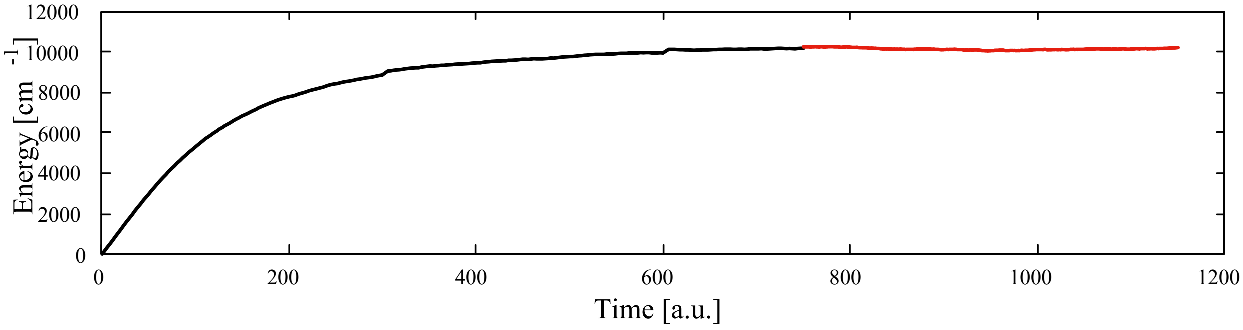


**Figure S3**. Time evolution of the DMC ensemble starting at time t = 0 with a delta-function distribution and an initial time size step Δt = 3 a.u. (~0.07 fs) followed by a smaller step 1 a.u. (0.02 fs) in the black part of the curve and reaching a steady state at t = 750 a.u. where the time size step is further reduced to 0.5 a.u. (~0.01 fs) in the red part of the curve. In all, $2.4*10^7$ configurations were generated in the steady state.

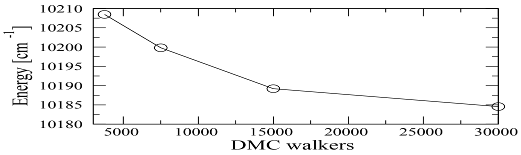


**Figure S4**. Convergence test of the ensemble energy as a function of the number of walkers with their corresponding energies averaged over several time snapshots within the steady state. One can see a well pronounced convergence with the number of walkers approaching 30000.

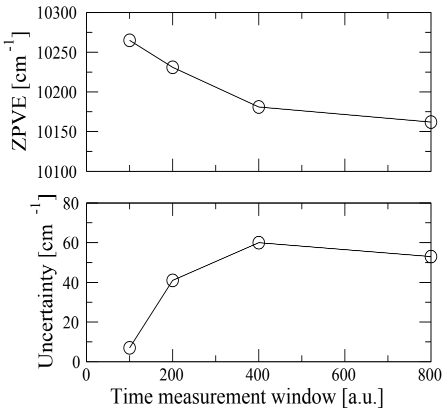


**Figure S5**. Convergence of the DMC ZPVE estimate and its uncertainty with respect to the time measurement window at the steady state. The present best estimate for ZPVE is 10162 ± 53 $cm^{-1}$ (or ± 0.5% of ZPVE). For comparison, the harmonic ZPVE is 10352 $cm^{-1}$.

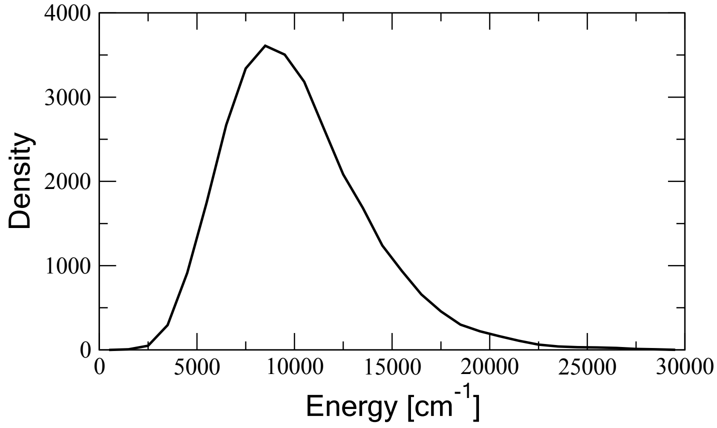


**Figure S6**. Distribution of the 30000 DMC energies at the final time step of the Monte Carlo simulation with 0.5 a.u. time step.

In Figure S6, we show the energy distribution at the end of the DMC simulation. The presently trained PES appears to be well behaved under the DMC conditions, in which we sampled $O(10^7)$ configurations and energies, many of which fall beyond the training set data. We note the

tail of the distribution extends to 25000 $cm^{-1}$ which is significantly higher than the highest energy in training set of ~16000 $cm^{-1}$.

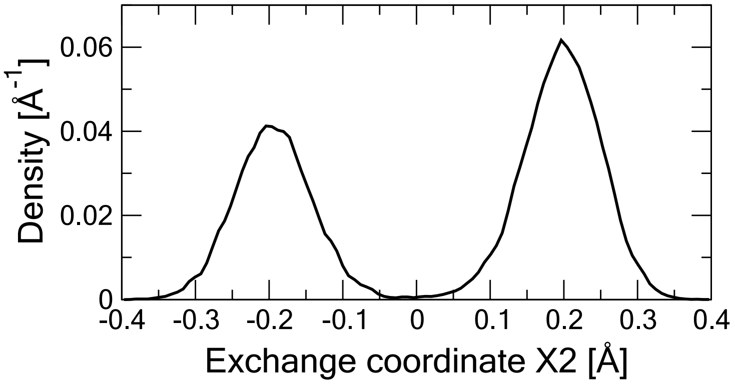


**Figure S7.** The final DMC density projected onto the exchange coordinate defined as the difference of the inner and outer radii, relative to the centroid, and averaged over the $C_{10}$ ring.

To conclude, we show the DMC configurations projected onto the ring deformation-inversion coordinate, seen in Figure S7. The density is slightly asymmetric, with roughly a 40-60% walker distribution between the two minima. The asymmetry is the result of the stochastic nature of the simulation and a finite propagation time. Clearly, at 0 Kelvin the vibrational wavefunction is localized entirely in the two $D_{5h}$ minima.

**S-5. The DVR calculations in 1-D**

Here, we estimate the temperature effect by calculating the wave functions and energies in 1-D using a Fourier grid DVR.[10] To use this approach, we define a rectilinear coordinate as the Cartesian vector connecting $D_{10h}$ and $D_{5h}$ equilibrium geometries (coordinate X1 used in Figure 1 and described in the main text), mass scale it, and diagonalize a small DVR matrix. This calculation reveals a picture similar to the DMC result but with an even more localized ground state density

at the $D_{5h}$ minimum. The first two states constitute a *gerade*/*ungerade* pair with the energies 200.8/200.9 $cm^{-1}$ relative to the $D_{5h}$ minimum, or ~611 $cm^{-1}$ below the $D_{10h}$ transition state, a very deep tunneling regime. The next pair of *gerade*/*ungerade* levels appear at 567.2/576.1 $cm^{-1}$, still well below the barrier energy, but nevertheless showing significant tunneling with a 0.3% probability of finding the *gerade* state at $D_{10h}$. The temperature corresponding to this level is ~528 K. A more systematic illustration of this is presented in Figure S8. We define probability density at temperature $T$ as a sum of the eigenstate densities weighted by the Boltzmann factor,

$$\rho_T(X1) = Z^{-1} \sum_{n=0} |\varphi_n(X1)|^2 exp\left[\left(E_n - E_0\right)/kT\right] \qquad (S3)$$

where $Z$ is the partition function. One can notice an emergence of $D_{10h}$ starting approximately at 300 K and becoming progressively viable at 500 and 1000 K.

**Table S3. Energy Levels in the 1-D Potential Describing the $D_{5h}$–$D_{10h}$–$D_{5h}$ Isomerization along the Cartesian Vector Connecting the Three Points. The Energies and Corresponding Wavefunctions were Calculated using a Fourier Grid DVR on a [-0.642, 0.642] Å Box with 100 intervals and a 0.013 Å Grid Spacing. Parity Labels g/u Stand for gerade/ungerade.**

| $n$ | parity | $E_n$ / $cm^{-1}$ | $E_n$ - $E_0$ / $cm^{-1}$ |
|---|---|---|---|
| 0 | g | 200.790 | 0 |
| 1 | u | 200.938 | 0.148 |
| 2 | g | 567.186 | 366.396 |
| 3 | u | 576.051 | 375.261 |
| 4 | g | 825.351 | 624.561 |
| 5 | u | 919.476 | 718.686 |
| 6 | g | 1105.222 | 904.432 |
| 7 | u | 1293.479 | 1092.689 |
| 8 | g | 1503.890 | 1303.100 |
| 9 | u | 1728.602 | 1527.812 |

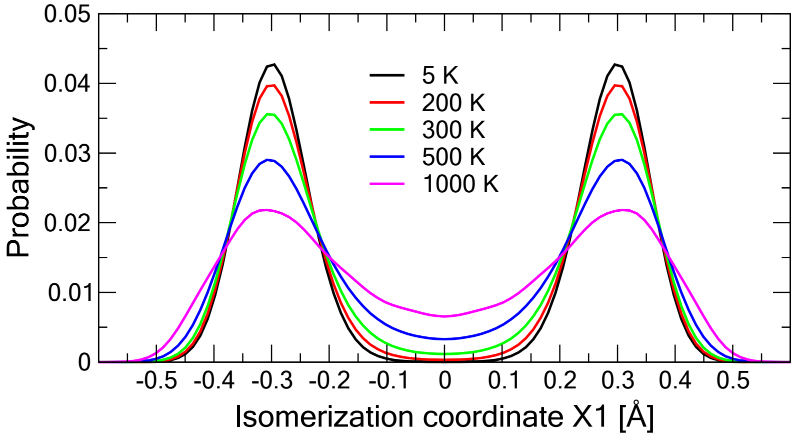


**Figure S8**. The DVR calculated probability distributions at various temperatures along the linear $D_{5h}$-$D_{10h}$ Cartesian isomerization coordinate X1. The DVR box is defined on [-0.642, 0.642] Å.

**References**

(1) Watts, J. D.; Bartlett, R. J. The nature of monocyclic $C_{10}$. A theoretical investigation using coupled-cluster methods. *Chem. Phys. Lett.* **1992**, *190*, 19-24.

(2) Braams, B. J.; Bowman, J. M. Permutationally Invariant Potential Energy Surfaces in High Dimensionality. *Int. Rev. Phys. Chem.* **2009**, *28*, 577-606.

(3) Qu, C.; Yu, Q.; Bowman, J. M. Permutationally Invariant Potential Energy Surfaces. *Annu. Rev. Phys. Chem.* **2018**, *69*, 151-175.

(4) Hashem, Y.; Foust, K.; Kaledin, M.; Kaledin, A. L. Fitting potential energy surfaces by learning the charge density matrix with permutationally invariant polynomials. *J. Chem. Theory Comput.* **2023**, *19*, 5690-5700.

(5) Karton, A.; Thimmakondu, V. S. CCSDT(Q)/CBS thermochemistry for the $D_{5h}$←→ $D_{10h}$ isomerization in the $C_{10}$ carbon cluster: Getting the right answer for the right reason. *Chem. Phys. Lett.* **2018**, *706*, 19-23.

(6) Xie, Z.; Bowman, J. M. Permutationally invariant polynomial basis for molecular energy surface fitting via monomial symmetrization. *J. Chem. Theory Comput.* **2010**, *6*, 26-34.

(7) Gaussian 16, Revision C.01, Frisch, M. J.; Trucks, G. W.; Schlegel, H. B.; Scuseria, G. E.; Robb, M. A.; Cheeseman, J. R.; Scalmani, G.; Barone, V.; Petersson, G. A.; Nakatsuji, H.; Li, X.; Caricato, M.; Marenich, A. V.; Bloino, J.; Janesko, B. G.; Gomperts, R.; Mennucci, B.; Hratchian, H. P.; Ortiz, J. V.; Izmaylov, A. F.; Sonnenberg, J. L.; Williams-Young, D.; Ding, F.; Lipparini, F.; Egidi, F.; Goings, J.; Peng, B.; Petrone, A.; Henderson, T.; Ranasinghe, D.; Zakrzewski, V. G.; Gao, J.; Rega, N.; Zheng, G.; Liang, W.; Hada, M.; Ehara, M.; Toyota, K.; Fukuda, R.; Hasegawa, J.; Ishida, M.; Nakajima, T.; Honda, Y.; Kitao, O.; Nakai, H.; Vreven, T.; Throssell, K.; Montgomery, J. A., Jr.; Peralta, J. E.; Ogliaro, F.; Bearpark, M. J.; Heyd, J. J.; Brothers, E. N.; Kudin, K. N.; Staroverov, V. N.; Keith, T. A.; Kobayashi, R.; Normand, J.; Raghavachari, K.; Rendell, A. P.; Burant, J. C.; Iyengar, S. S.; Tomasi, J.; Cossi, M.; Millam, J. M.; Klene, M.; Adamo, C.; Cammi, R.; Ochterski, J. W.; Martin, R. L.; Morokuma, K.; Farkas, O.; Foresman, J. B.; Fox, D. J. Gaussian, Inc., Wallingford CT, 2016.

(8) MOLPRO, version 2022.2, a Package of *Ab Initio* Programs. Werner, H.-J.; Knowles, P. J.; Knizia, G.; Manby, F. R.; Schütz, M.; Celani, P.; Györffy, W.; Kats, D.; Korona, T.; Lindh, R.; Mitrushenkov, A.; Rauhut, G.; Shamasundar, K. R.; Adler, T. B.; Amos, R. D.; Bennie, S. J.; Bernhardsson, A.; Berning, A.; Cooper, D. L.; Deegan, M. J. O.; Dobbyn, A. J.; Eckert, F.; Goll, E.; Hampel, C.; Hesselmann, A.; Hetzer, G.; Hrenar, T.; Jansen, G.; Köppl, C.; Lee, S. J. R.; Liu, Y.; Lloyd, A. W.; Ma, Q.; Mata, R. A.; May, A. J.; McNicholas, S. J.; Meyer, W.; Miller III, T.

F.; Mura, M. E.; Nicklass, A.; O'Neill, D. P.; Palmieri, P.; Peng, D. ; Pflüger, K.; Pitzer, R.; Reiher, M.; Shiozaki, T.; Stoll, H.; Stone, A. J.; Tarroni, R.; Thorsteinsson, T.; Wang, M.; Welborn, M. https://www.molpro.net.

(9) Anderson, J. B. A random-walk simulation of the Schrodinger equation: $H_3^+$. *J. Chem. Phys.* **1975**, *63*, 1499-1503.

(10) Colbert, D. T.; Miller, W. H. A novel discrete variable representation for quantum mechanical reactive scattering via the S-matrix Kohn method. J. Chem. Phys. **1991**, *96*, 1982-1991.
