# Supplementary material for: Quantum Monte Carlo Simulations of the Vibrational Wavefunction of the Aromatic Cyclo[10]carbon Using a Full Dimensional Permutationally Invariant Potential Energy Surface
Source: J Phys Chem Lett. 2024 May 3;15(19):5070–5. doi: 10.1021/acs.jpclett.4c00893 (PMC11103689; doi:10.1021/acs.jpclett.4c00893)
Supplement: Supplementary file 2 — jz4c00893_si_002.pdf [file jz4c00893_si_002.pdf]

jz-2024-00893a.R1

Name: Peer Review Information for "Quantum Monte Carlo Simulations of the Vibrational Wavefunction of the Aromatic Cyclo[10]Carbon Using a Full Dimensional Permutationally Invariant Potential Energy Surface"

First Round of Reviewer Comments

Reviewer: 1

Comments to the Author

1. What is the major advance reported in the paper?

This paper reports a new theory study of the C10 molecule by solving the vibration wavefunction using quantum Monte Carlo. C10 is a typical aromatic molecule, which has a debated structure theoretically for a long period. The difficulty of the problem stems from the sensitivity of electronic structure and the vibrational dynamics, i.e. nuclear quantum effects. To confirm the stable structure computationally, one needs to treat the electronic structure at high level theory and at the same time treat the nuclear quantum effects. Therefore, in previous theoretical works, one approximated treatment may lead to a different result from a different approximation. Recent theories were leaning towards the symmetry D_10h octagon structure, but recent an experiment provided evidence for a cumulenic D_5h pentagon structure. This work provides the latest and the most accurate theoretical description using quantum Monte Carlo to treat nuclear quantum effects. Their calculations represent a reasonable explanation of the experimental findings. Although in previous theoretical works, the same answer may also be reached with approximated methods. I think getting the right answer with the right method is very valuable.

2. What is the immediate significance of this advance?

The immediate significance is to provide a reference calculation for those who are interested in developing theoretical methods to study similar systems.

3. Technical suggestions

Since the current work is based on the PES at the same level as ref. 16, the main difference is the treatment of nuclear quantum effects. In this work, the use of QMC is expected to be superior to the simple estimate of ZPE in ref. 16. I hope the authors can provide more discussion on why ZPE estimates are less reliable in this particular system, which might be insightful for people who are

interested in estimating nuclear quantum effects but can not afford to do QMC calculations for every system.

As QMC is a stochastic method, I would suggest the authors provide estimates of statistical errors. From Fig. S3, it is not clear to me whether the results are fully converged (with negligible statistical error) or not. I would also like to see a confirmation of the convergence as a function of the number of walkers and the time-step. In my opinion the meaning of this study is more on the quantitative side, so I would think reporting high-quality well-converged values with proper estimates of errors is valuable to the community.

Reviewer: 2

Comments to the Author

In this paper, Gibbas, Kaledin and Kaledin tackle an open problem in vibrational spectroscopy and structural characterization of molecular systems: the case of C₁₀. There seemed to be general agreement about a D_{10h} average structure for C₁₀ . However, this was recently questioned by a new experiment showing a D_{5h} equilibrium structure for the molecule.

The major advance of this paper is represented by the fact that the authors demonstrate by means of state-of-art calculations that the ground state structure of C₁₀ has indeed a prevalent D_{5h} character (about 96%). In this way they were able to settle the long on-going controversy corroborating the latest experimental data available.

The immediate significance of the paper, in addition to the solution to the open problem, lies in the demonstration that to solve this type of very refined open issues it is necessary to employ both state-of-art electronic theory calculations and full-dimensional vibrational investigations.

From the technical point of view, the authors employ their previously developed method in the framework of the permutationally invariant polynomial approach to build potential energy surfaces (PESs). Furthermore, they adopt full-dimensional diffusion Monte Carlo (DMC) calculations to determine the ground state eigenfunction.

In my opinion this interesting manuscript is suitable for publication in the Journal of Physical Chemistry Letters upon revision based on these few comments:

1) I think the mathematical expression of the morphing function should be reported in the main part of the manuscript and not only in the Supplementary Information file.

2) On pp. 15-16 it is not clear what the authors mean by “normalized sum” in computing the C-C spectrum. Did they just divide the number of C-C distances collected in any bin by the total number of Monte Carlo configurations? Were they doing something different? A more detailed text is maybe needed on this point.

3) As a general possibility of improvement for the authors’ PIP PES technique, automatic differentiation has recently been coded into PIP PESs. Could the authors take advantage of the same technique and implement a better differentiation/gradient routine in their software?

Author's Response to Peer Review Comments:

We thank both reviewers for their valuable advice and encouragment to expand the discussion. The original reviewer queries appear in black. **All of our responses are in bold.** The actual text added in the submitted documents is in red and enclosed in " ".

Reviewer(s)' Comments to Author:

Reviewer: 1

Recommendation: This paper is probably publishable, but major revision is needed; I do not need to see future revisions.

Comments:

1. What is the major advance reported in the paper?

This paper reports a new theory study of the C10 molecule by solving the vibration wavefunction using quantum Monte Carlo. C10 is a typical aromatic molecule, which has a debated structure theoretically for a long period. The difficulty of the problem stems from the sensitivity of electronic structure and the vibrational dynamics, i.e. nuclear quantum effects. To confirm the stable structure computationally, one needs to treat the electronic structure at high level theory and at the same time treat the nuclear quantum effects. Therefore, in previous theoretical works, one approximated treatment may lead to a different result from a different approximation. Recent theories were leaning towards the symmetry D_10h octagon structure, but recent an experiment provided evidence for a cumulenic D_5h pentagon structure. This work provides the latest and the most accurate theoretical description using quantum Monte Carlo to treat nuclear quantum effects. Their calculations represent a reasonable explanation of the experimental findings. Although in previous theoretical works, the same answer may also be reached with approximated methods. I think getting the right answer with the right method is very valuable.

2. What is the immediate significance of this advance?

The immediate significance is to provide a reference calculation for those who are interested in developing theoretical methods to study similar systems.

3. Technical suggestions

Since the current work is based on the PES at the same level as ref. 16, the main difference is the treatment of nuclear quantum effects. In this work, the use of QMC is expected to be superior to the simple estimate of ZPE in ref. 16. I hope the authors can provide more discussion on why ZPE estimates are less reliable in this particular system, which might be insightful for people who are interested in estimating nuclear quantum effects but can not afford to do QMC calculations for every system.

It is important to distinguish between the harmonic/Hessian-based ZPVE treatment and full variational quantum treatment of the vibrational wavefunction. To accomplish the latter, it is imperative to have a well-trained analytic PES, which is what we have done in this work. To better stress these points, we rephrased a key sentence on page 3 as shown,

"The authors also found that the addition of the ZPVE at the harmonic level, albeit with B3LYP/cc-pVTZ..."

and slightly expanded description of our aims on page 5 of main text,

"on an analytic potential energy surface (PES)"

As QMC is a stochastic method, I would suggest the authors provide estimates of statistical errors. From Fig. S3, it is not clear to me whether the results are fully converged (with negligible statistical error) or not.

These are excellent suggestions. We have carried out additional analyses of our DMC data and addressed these questions in the SI and the main text.

The old Figure S3 shows only a small part of the DMC simulation after the DMC ensemble has reached a steady state, identified as a state of fluctuating energy about an average. We have clarified this point by adding data and replacing old Figure S3 with a new one which shows a clear convergence of the energy from the initial stages (black colored data) of the simulation to the steady state (red colored data). In addition to the average of the energy in the steady state, which we report as out best ZPVE estimate (10162 cm^{-1}), we are also reporting the uncertainty in the statistical sample (53 cm^{-1}), i.e. the statistical error estimate of the ZPVE as requested by the reviewer. The uncertainty constitutes about 0.5% of the total ZPVE value. These values are supplied in the new Figure S5 caption as well as in the main text on page 14:

"which in the present DMC simulations corresponds to the ground state vibrational energy (ZPVE) of 10162 ± 53 cm^{-1}."

We also added text on page S6 of the SI,

"followed by additional statistical data in Figure S4 and Figure S5."

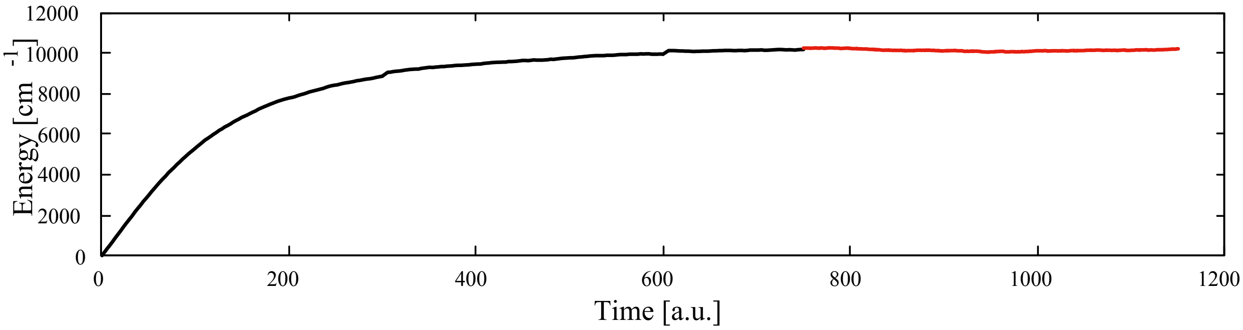

Figure S3 (NEW). Time evolution of the DMC ensemble starting at time t = 0 with a delta-function distribution and an initial time size step Δt = 3 a.u. (~0.07 fs) followed by a smaller step 1 a.u. (0.02 fs) in the black part of the curve and reaching a steady state at t = 750 a.u. where the time size step is further reduced to 0.5 a.u. (~0.01 fs) in the red part of the curve. In all, 2.4*10^7 configurations were generated in the steady state.

I would also like to see a confirmation of the convergence as a function of the number of walkers and the time-step. In my opinion the meaning of this study is more on the quantitative side, so I would think reporting high-quality well-converged values with proper estimates of errors is valuable to the community.

Duly noted and appreciated. We provide the requested evidence in the new Figure S4, which shows a test of convergence of the energy with respect to the number of walkers for several selected time snapshots within the steady state.

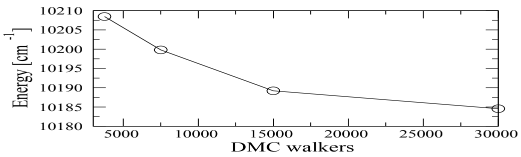

Figure S4 (NEW). Convergence test of the ensemble energy as a function of the number of walkers with their corresponding energies averaged over several time snapshots within the steady state. One can see a well pronounced convergence with the number of walkers approaching 30000.

As suggested by the reviewer, evidence of convergence with respect to propagation time is shown in the new Figure S5 which shows convergence of the ZPVE estimate and the uncertainty estimate with respect to the time-measurement window in the steady state.

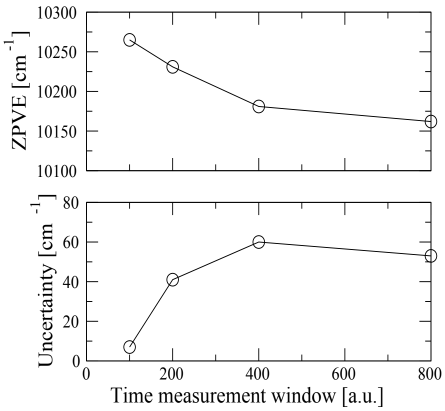

Figure S5 (NEW). Convergence of the DMC ZPVE estimate and its uncertainty with respect to the time measurement window at the steady state. The present best estimate for ZPVE is 10162 ± 53 cm^{-1} (or ± 0.5% of ZPVE). For comparison, the harmonic ZPVE is 10352 cm^{-1}.

Additional Questions:
Urgency: Top 10%

Significance: Top 10%

Novelty: Top 10%

Scholarly Presentation: Top 10%

Is the paper likely to interest a substantial number of physical chemists, not just specialists working in the authors' area of research?: Yes

Reviewer: 2

Recommendation: This paper is publishable subject to minor revisions noted. Further review is not needed.

Comments:
In this paper, Gibbas, Kaledin and Kaledin tackle an open problem in vibrational spectroscopy and structural characterization of molecular systems: the case of C₁₀. There seemed to be general agreement about a D_{10h} average structure for C₁₀ . However, this was recently questioned by a new experiment showing a D_{5h} equilibrium structure for the molecule.

The major advance of this paper is represented by the fact that the authors demonstrate by means of state-of-art calculations that the ground state structure of C₁₀ has indeed a prevalent D_{5h} character (about 96%). In this way they were able to settle the long on-going controversy corroborating the latest experimental data available.

The immediate significance of the paper, in addition to the solution to the open problem, lies in the demonstration that to solve this type of very refined open issues it is necessary to employ both state-of-art electronic theory calculations and full-dimensional vibrational investigations.

From the technical point of view, the authors employ their previously developed method in the framework of the permutationally invariant polynomial approach to build potential energy surfaces (PESs). Furthermore, they adopt full-dimensional diffusion Monte Carlo (DMC) calculations to determine the ground state eigenfunction.

In my opinion this interesting manuscript is suitable for publication in the Journal of Physical Chemistry Letters upon revision based on these few comments:

1) I think the mathematical expression of the morphing function should be reported in the main part of the manuscript and not only in the Supplementary Information file.

We have considered the reviewer's suggestion and moved this equation to the main text as the new Eq. 2 along with moving the descriptive narrative from the SI onto page 9 of the MS, as follows,

"...and use the following morphing function

$$V_{\tau\mathrm{HCTH-CC}} = \frac{w\Delta E_{\mathrm{CC}} + [1-w]\Delta E_{\tau\mathrm{HCTH}}}{\Delta E_{\tau\mathrm{HCTH}}} V_{\tau\mathrm{HCTH}} \qquad (2)$$

where the zero of the $V_{\tau\mathrm{HCTH}}$ energy is set at the D_{10h} transition state, ΔE_{CC} is the benchmark (CC = CCSDT(Q)/CBS) barrier height,[16] $\Delta E_{\tau\mathrm{HCTH}}$ is the τHCTH barrier height and the energy-dependent weight factor is

$$w = exp\left[-\left(\frac{V_{\tau\mathrm{HCTH}} + \Delta E_{\tau\mathrm{HCTH}}}{\Delta E_{\mathrm{CC}}}\right)^3\right] \qquad (3)$$

For the geometries where $V_{\tau\mathrm{HCTH}}$ is slightly above the minimum, that is where $V_{\tau\mathrm{HCTH}} \approx -\Delta E_{\tau\mathrm{HCTH}}$, we have $w \approx 1$, and therefore $V_{\tau\mathrm{HCTH-CC}} \approx (\Delta E_{\mathrm{CC}}/\Delta E_{\tau\mathrm{HCTH}})V_{\tau\mathrm{HCTH}}$, a simple scalar modification of the original τHCTH data. At moderate and much higher energies, $w \rightarrow 0$ very rapidly, given the cubed exponential, and $V_{\tau\mathrm{HCTH}\rightarrow\mathrm{CC}} \approx V_{\tau\mathrm{HCTH}}$, i.e., remains unmodified from the τHCTH data. We also point out that the ratio $\Delta E_{\mathrm{CC}}/\Delta E_{\tau\mathrm{HCTH}}$ should not deviate significantly from unity to avoid introducing artificial lobs and valleys in the modified energy landscape, which can be readily seen by analyzing Eq. 2 in one dimension. Thus the present choice of τHCTH/cc-pVQZ for the source data is well justified (cf. $\Delta\Delta E$ values in Table 1)."

2) On pp. 15-16 it is not clear what the authors mean by "normalized sum" in computing the C-C spectrum. Did they just divide the number of C-C distances collected in any bin by the total number of Monte Carlo configurations? Were they doing something different? A more detailed text is maybe needed on this point.

We thank the reviewer for catching the confusing wording. The text on page 13 of the MS has been modified accordingly as follows:

"...by measuring each of the 45 internuclear distances at these configurations and binning them on the [1, 4.5] Å range."

3) As a general possibility of improvement for the authors' PIP PES technique, automatic differentiation has recently been coded into PIP PESs. Could the authors take advantage of the same technique and implement a better differentiation/gradient routine in their software?

This is a valuable observation that points to a potentially very efficient development of the CHM method described here for (semi)classical MD simulations. We add the following reference (new Ref 34) to reflect this possibility along with corresponding text,

"In perspective, however, and as noted recently by method development groups,[34] analytic gradients of PIP based PESs may be readily computed by the PESPIP software."

(34) Houston, P. L.; Qu, C.; Yu, Q.; Conte, R,; Nandi, A.; Li, J. K.; Bowman, J. M. PESPIP: Software to fit complex molecular and many-body potential energy surfaces with permutationally invariant polynomials. *J. Chem. Phys.* **2023**, *158*, 044109.

Additional Questions:
Urgency: High

Significance: Top 10%

Novelty: High

Scholarly Presentation: High

Is the paper likely to interest a substantial number of physical chemists, not just specialists working in the authors' area of research?: Yes
